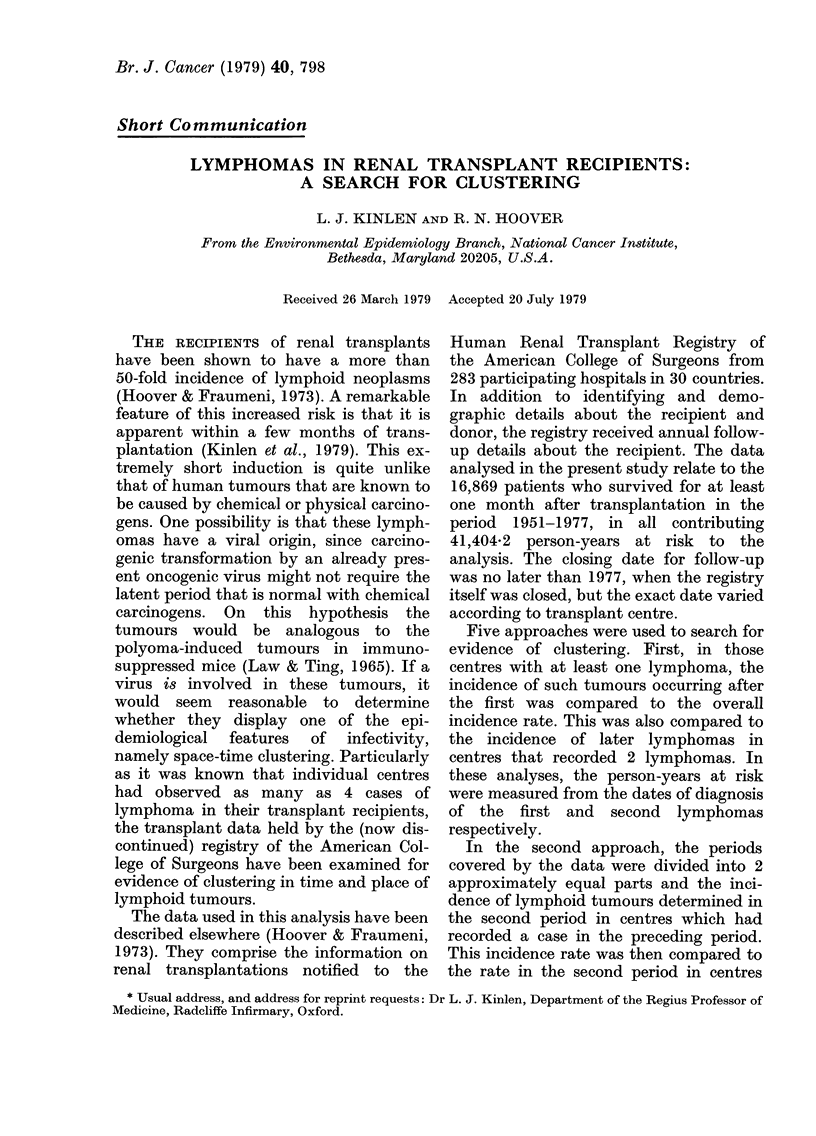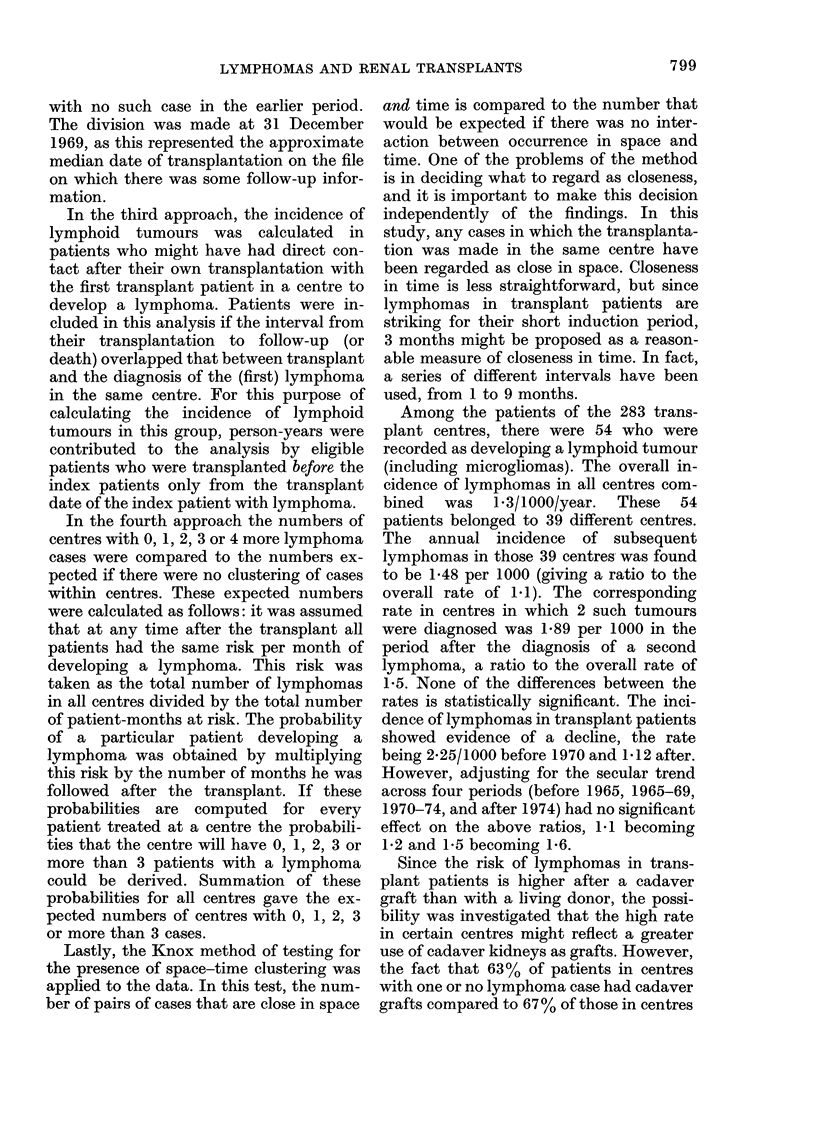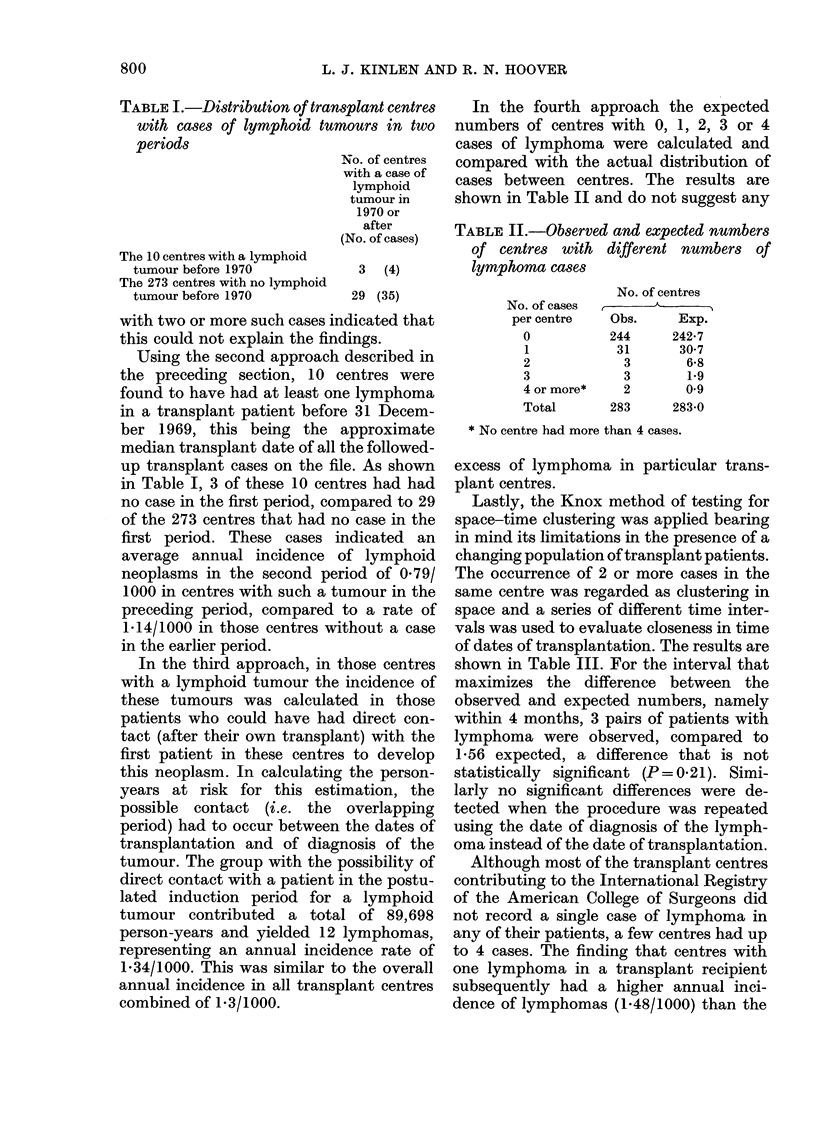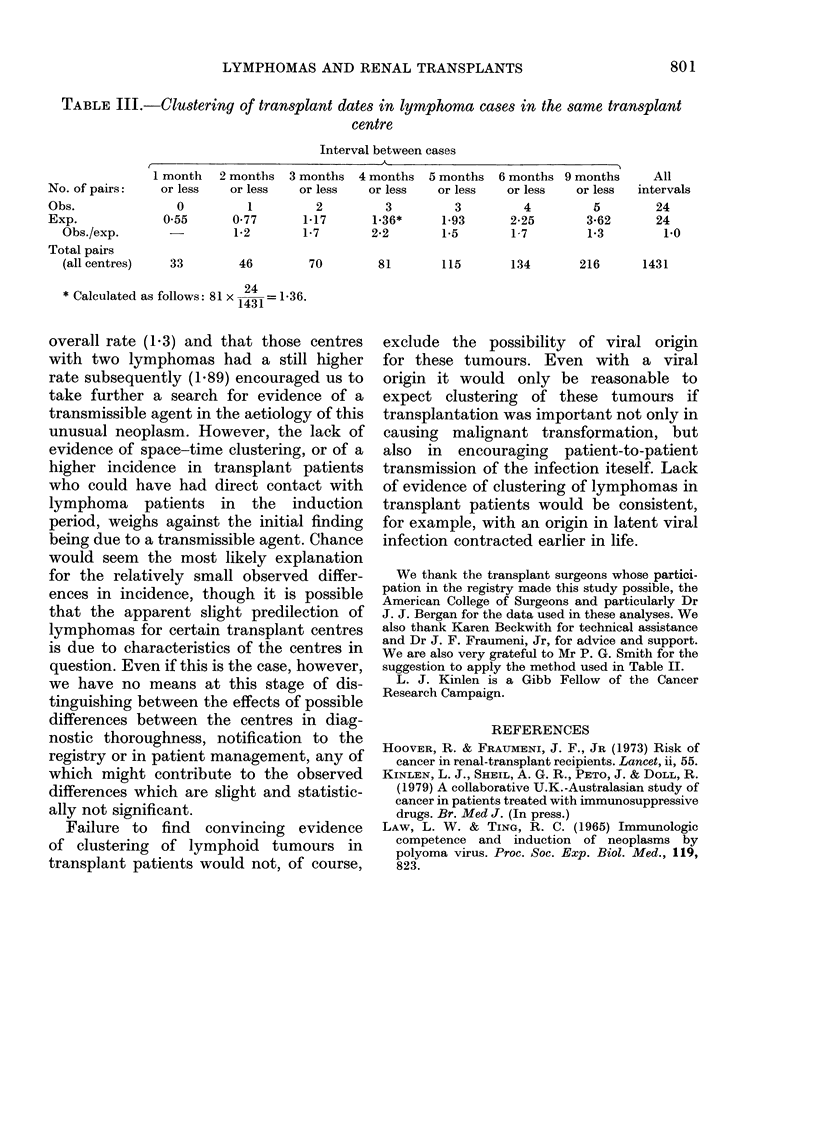# Lymphomas in renal transplant recipients: a search for clustering.

**DOI:** 10.1038/bjc.1979.263

**Published:** 1979-11

**Authors:** L. J. Kinlen, R. N. Hoover


*Br. J. Cancer* (1979) **40**, 798

*Short Communication*

### LYMPHOMAS IN RENAL TRANSPLANT RECIPIENTS: A SEARCH FOR CLUSTERING

L. J. KINLEN AND R. N. HOOVER

*From the Environmental Epidemiology Branch, National Cancer Institute, Bethesda, Maryland* 20205, *U.S.A.*

Received 26 March 1979 Accepted 20 July 1979

THE RECIPIENTS of renal transplants have been shown to have a more than 50-fold incidence of lymphoid neoplasms (Hoover & Fraumeni, 1973). A remarkable feature of this increased risk is that it is apparent within a few months of transplantation (Kinlen *et al.*, 1979). This extremely short induction is quite unlike that of human tumours that are known to be caused by chemical or physical carcinogens. One possibility is that these lymphomas have a viral origin, since carcinogenic transformation by an already present oncogenic virus might not require the latent period that is normal with chemical carcinogens. On this hypothesis the tumours would be analogous to the polyoma-induced tumours in immunosuppressed mice (Law & Ting, 1965). If a virus *is* involved in these tumours, it would seem reasonable to determine whether they display one of the epidemiological features of infectivity, namely space-time clustering. Particularly as it was known that individual centres had observed as many as 4 cases of lymphoma in their transplant recipients, the transplant data held by the (now discontinued) registry of the American College of Surgeons have been examined for evidence of clustering in time and place of lymphoid tumours.

The data used in this analysis have been described elsewhere (Hoover & Fraumeni, 1973). They comprise the information on renal transplantations notified to the Human Renal Transplant Registry of the American College of Surgeons from 283 participating hospitals in 30 countries. In addition to identifying and demographic details about the recipient and donor, the registry received annual follow-up details about the recipient. The data analysed in the present study relate to the 16,869 patients who survived for at least one month after transplantation in the period 1951–1977, in all contributing 41,404·2 person-years at risk to the analysis. The closing date for follow-up was no later than 1977, when the registry itself was closed, but the exact date varied according to transplant centre.

Five approaches were used to search for evidence of clustering. First, in those centres with at least one lymphoma, the incidence of such tumours occurring after the first was compared to the overall incidence rate. This was also compared to the incidence of later lymphomas in centres that recorded 2 lymphomas. In these analyses, the person-years at risk were measured from the dates of diagnosis of the first and second lymphomas respectively.

In the second approach, the periods covered by the data were divided into 2 approximately equal parts and the incidence of lymphoid tumours determined in the second period in centres which had recorded a case in the preceding period. This incidence rate was then compared to the rate in the second period in centres

* Usual address, and address for reprint requests: Dr L. J. Kinlen, Department of the Regius Professor of Medicine, Radcliffe Infirmary, Oxford.

with no such case in the earlier period. The division was made at 31 December 1969, as this represented the approximate median date of transplantation on the file on which there was some follow-up information.

In the third approach, the incidence of lymphoid tumours was calculated in patients who might have had direct contact after their own transplantation with the first transplant patient in a centre to develop a lymphoma. Patients were included in this analysis if the interval from their transplantation to follow-up (or death) overlapped that between transplant and the diagnosis of the (first) lymphoma in the same centre. For this purpose of calculating the incidence of lymphoid tumours in this group, person-years were contributed to the analysis by eligible patients who were transplanted *before* the index patients only from the transplant date of the index patient with lymphoma.

In the fourth approach the numbers of centres with 0, 1, 2, 3 or 4 more lymphoma cases were compared to the numbers expected if there were no clustering of cases within centres. These expected numbers were calculated as follows: it was assumed that at any time after the transplant all patients had the same risk per month of developing a lymphoma. This risk was taken as the total number of lymphomas in all centres divided by the total number of patient-months at risk. The probability of a particular patient developing a lymphoma was obtained by multiplying this risk by the number of months he was followed after the transplant. If these probabilities are computed for every patient treated at a centre the probabilities that the centre will have 0, 1, 2, 3 or more than 3 patients with a lymphoma could be derived. Summation of these probabilities for all centres gave the expected numbers of centres with 0, 1, 2, 3 or more than 3 cases.

Lastly, the Knox method of testing for the presence of space–time clustering was applied to the data. In this test, the number of pairs of cases that are close in space *and* time is compared to the number that would be expected if there was no interaction between occurrence in space and time. One of the problems of the method is in deciding what to regard as closeness, and it is important to make this decision independently of the findings. In this study, any cases in which the transplantation was made in the same centre have been regarded as close in space. Closeness in time is less straightforward, but since lymphomas in transplant patients are striking for their short induction period, 3 months might be proposed as a reasonable measure of closeness in time. In fact, a series of different intervals have been used, from 1 to 9 months.

Among the patients of the 283 transplant centres, there were 54 who were recorded as developing a lymphoid tumour (including microgliomas). The overall incidence of lymphomas in all centres combined was 1·3/1000/year. These 54 patients belonged to 39 different centres. The annual incidence of subsequent lymphomas in those 39 centres was found to be 1·48 per 1000 (giving a ratio to the overall rate of 1·1). The corresponding rate in centres in which 2 such tumours were diagnosed was 1·89 per 1000 in the period after the diagnosis of a second lymphoma, a ratio to the overall rate of 1·5. None of the differences between the rates is statistically significant. The incidence of lymphomas in transplant patients showed evidence of a decline, the rate being 2·25/1000 before 1970 and 1·12 after. However, adjusting for the secular trend across four periods (before 1965, 1965–69, 1970–74, and after 1974) had no significant effect on the above ratios, 1·1 becoming 1·2 and 1·5 becoming 1·6.

Since the risk of lymphomas in transplant patients is higher after a cadaver graft than with a living donor, the possibility was investigated that the high rate in certain centres might reflect a greater use of cadaver kidneys as grafts. However, the fact that 63% of patients in centres with one or no lymphoma case had cadaver grafts compared to 67% of those in centres

TABLE I.—*Distribution of transplant centres with cases of lymphoid tumours in two periods*

| | No. of centres with a case of lymphoid tumour in 1970 or after (No. of cases) |
|---|---|
| The 10 centres with a lymphoid tumour before 1970 | 3 (4) |
| The 273 centres with no lymphoid tumour before 1970 | 29 (35) |

with two or more such cases indicated that this could not explain the findings.

Using the second approach described in the preceding section, 10 centres were found to have had at least one lymphoma in a transplant patient before 31 December 1969, this being the approximate median transplant date of all the followed-up transplant cases on the file. As shown in Table I, 3 of these 10 centres had had no case in the first period, compared to 29 of the 273 centres that had no case in the first period. These cases indicated an average annual incidence of lymphoid neoplasms in the second period of 0·79/1000 in centres with such a tumour in the preceding period, compared to a rate of 1·14/1000 in those centres without a case in the earlier period.

In the third approach, in those centres with a lymphoid tumour the incidence of these tumours was calculated in those patients who could have had direct contact (after their own transplant) with the first patient in these centres to develop this neoplasm. In calculating the person-years at risk for this estimation, the possible contact (*i.e.* the overlapping period) had to occur between the dates of transplantation and of diagnosis of the tumour. The group with the possibility of direct contact with a patient in the postulated induction period for a lymphoid tumour contributed a total of 89,698 person-years and yielded 12 lymphomas, representing an annual incidence rate of 1·34/1000. This was similar to the overall annual incidence in all transplant centres combined of 1·3/1000.

In the fourth approach the expected numbers of centres with 0, 1, 2, 3 or 4 cases of lymphoma were calculated and compared with the actual distribution of cases between centres. The results are shown in Table II and do not suggest any excess of lymphoma in particular transplant centres.

TABLE II.—*Observed and expected numbers of centres with different numbers of lymphoma cases*

| No. of cases per centre | No. of centres Obs. | Exp. |
|---|---|---|
| 0 | 244 | 242·7 |
| 1 | 31 | 30·7 |
| 2 | 3 | 6·8 |
| 3 | 3 | 1·9 |
| 4 or more* | 2 | 0·9 |
| Total | 283 | 283·0 |

* No centre had more than 4 cases.

Lastly, the Knox method of testing for space–time clustering was applied bearing in mind its limitations in the presence of a changing population of transplant patients. The occurrence of 2 or more cases in the same centre was regarded as clustering in space and a series of different time intervals was used to evaluate closeness in time of dates of transplantation. The results are shown in Table III. For the interval that maximizes the difference between the observed and expected numbers, namely within 4 months, 3 pairs of patients with lymphoma were observed, compared to 1·56 expected, a difference that is not statistically significant ($P = 0{\cdot}21$). Similarly no significant differences were detected when the procedure was repeated using the date of diagnosis of the lymphoma instead of the date of transplantation.

Although most of the transplant centres contributing to the International Registry of the American College of Surgeons did not record a single case of lymphoma in any of their patients, a few centres had up to 4 cases. The finding that centres with one lymphoma in a transplant recipient subsequently had a higher annual incidence of lymphomas (1·48/1000) than the

TABLE III.—*Clustering of transplant dates in lymphoma cases in the same transplant centre*

| No. of pairs: | Interval between cases: 1 month or less | 2 months or less | 3 months or less | 4 months or less | 5 months or less | 6 months or less | 9 months or less | All intervals |
|---|---|---|---|---|---|---|---|---|
| Obs. | 0 | 1 | 2 | 3 | 3 | 4 | 5 | 24 |
| Exp. | 0·55 | 0·77 | 1·17 | 1·36* | 1·93 | 2·25 | 3·62 | 24 |
| Obs./exp. | — | 1·2 | 1·7 | 2·2 | 1·5 | 1·7 | 1·3 | 1·0 |
| Total pairs (all centres) | 33 | 46 | 70 | 81 | 115 | 134 | 216 | 1431 |

* Calculated as follows: $81 \times \frac{24}{1431} = 1{\cdot}36$.

overall rate (1·3) and that those centres with two lymphomas had a still higher rate subsequently (1·89) encouraged us to take further a search for evidence of a transmissible agent in the aetiology of this unusual neoplasm. However, the lack of evidence of space–time clustering, or of a higher incidence in transplant patients who could have had direct contact with lymphoma patients in the induction period, weighs against the initial finding being due to a transmissible agent. Chance would seem the most likely explanation for the relatively small observed differences in incidence, though it is possible that the apparent slight predilection of lymphomas for certain transplant centres is due to characteristics of the centres in question. Even if this is the case, however, we have no means at this stage of distinguishing between the effects of possible differences between the centres in diagnostic thoroughness, notification to the registry or in patient management, any of which might contribute to the observed differences which are slight and statistically not significant.

Failure to find convincing evidence of clustering of lymphoid tumours in transplant patients would not, of course, exclude the possibility of viral origin for these tumours. Even with a viral origin it would only be reasonable to expect clustering of these tumours if transplantation was important not only in causing malignant transformation, but also in encouraging patient-to-patient transmission of the infection iteself. Lack of evidence of clustering of lymphomas in transplant patients would be consistent, for example, with an origin in latent viral infection contracted earlier in life.

We thank the transplant surgeons whose participation in the registry made this study possible, the American College of Surgeons and particularly Dr J. J. Bergan for the data used in these analyses. We also thank Karen Beckwith for technical assistance and Dr J. F. Fraumeni, Jr, for advice and support. We are also very grateful to Mr P. G. Smith for the suggestion to apply the method used in Table II.

L. J. Kinlen is a Gibb Fellow of the Cancer Research Campaign.

### REFERENCES

HOOVER, R. & FRAUMENI, J. F., JR (1973) Risk of cancer in renal-transplant recipients. *Lancet*, ii, 55.

KINLEN, L. J., SHEIL, A. G. R., PETO, J. & DOLL, R. (1979) A collaborative U.K.-Australasian study of cancer in patients treated with immunosuppressive drugs. *Br. Med J.* (In press.)

LAW, L. W. & TING, R. C. (1965) Immunologic competence and induction of neoplasms by polyoma virus. *Proc. Soc. Exp. Biol. Med.*, **119**, 823.